# Right precordial-directed electrocardiographical markers identify arrhythmogenic right ventricular cardiomyopathy in the absence of conventional depolarization or repolarization abnormalities

**DOI:** 10.1186/s12872-017-0696-x

**Published:** 2017-10-13

**Authors:** Daniel Cortez, Anneli Svensson, Jonas Carlson, Sharon Graw, Nandita Sharma, Francesca Brun, Anita Spezzacatene, Luisa Mestroni, Pyotr G. Platonov

**Affiliations:** 1 Department of Cardiology, Clinical Sciences, Lund University, Lund, Sweden; 2 Electrophysiology/Cardiology, Penn State Milton S. Hershey Medical Center, Hershey, USA; 3 Department of Cardiology and Department of Medical and Health Sciences, Linkoping University, Linkoping, Sweden; 4 Cardiovascular Institute and Adult Medical Genetics Program, University of Colorado Denver AMC, Aurora, CO USA; 5 Cardiovascular Department, Ospedali Riuniti and University of Trieste, Trieste, Italy; 6 Arrhythmia Clinic, Skåne University Hospital, Lund, Sweden

**Keywords:** Arrhythmogenic right ventricular cardiomyopathy, Vectorcardiography, ECG, Cascade screening

## Abstract

**Background:**

Arrhythmogenic right ventricular dysplasia/cardiomyopathy (ARVD/C) carries a risk of sudden death. We aimed to assess whether vectorcardiographic (VCG) parameters directed toward the right heart and a measured angle of the S-wave would help differentiate ARVD/C with otherwise normal electrocardiograms from controls.

**Methods:**

Task Force 2010 definite ARVD/C criteria were met for all patients. Those who did not fulfill Task Force depolarization or repolarization criteria (−ECG) were compared with age and gender-matched control subjects. Electrocardiogram measures of a 3-dimentional spatial QRS-T angle, a right-precordial-directed orthogonal QRS-T (RPD) angle, a root mean square of the right sided depolarizing forces (RtRMS-QRS), QRS duration (QRSd) and the corrected QT interval (QTc), and a measured angle including the upslope and downslope of the S-wave (S-wave angle) were assessed.

**Results:**

Definite ARVD/C was present in 155 patients by 2010 Task Force criteria (41.7 ± 17.6 years, 65.2% male). -ECG ARVD/C patients (66 patients) were compared to 66 control patients (41.7 ± 17.6 years, 65.2% male). All parameters tested except the QRSd and QTc significantly differentiated -ECG ARVD/C from control patients (*p* < 0.004 to *p* < 0.001). The RPD angle and RtRMS-QRS best differentiated the groups. Combined, the 2 novel criteria gave 81.8% sensitivity, 90.9% specificity and odds ratio of 45.0 (95% confidence interval 15.8 to 128.2).

**Conclusion:**

ARVD/C disease process may lead to development of subtle ECG abnormalities that can be distinguishable using right-sided VCG or measured angle markers better than the spatial QRS-T angle, the QRSd or QTc, in the absence of Taskforce ECG criteria.

## Background

Arrhythmogenic right ventricular dysplasia/cardiomyopathy (ARVD/C) is an inherited cardiomyopathy characterized by fibro-fatty replacement of predominately the right ventricle, which predisposes patients to life-threatening ventricular arrhythmias and usually slowly progressive ventricular dysfunction [1]. The disease is inherited as an autosomal dominant trait with incomplete penetrance and highly variable expressivity [1]. Diagnosis is made by combining multiple sources of diagnostic information as prescribed by the Task Force criteria, which were updated in 2010 to increase diagnostic sensitivity while maintaining specificity [2].

First-degree relatives often have incomplete expression of the disease [3]. Clinical cascade screening of family members in genotype-negative ARVD/C is complicated by the lack of early specific signs of disease that would identify those individuals prone to development of disease. Electrocardiographic (ECG) changes may develop before histologic evidence of myocyte loss or clinical evidence of RV dysfunction [4, 5]. However, ECG depolarization and repolarization changes, based on current criteria, are typically only apparent in around half of family members who eventually progress to meet Definite ARVD/C by 2010 criteria [5].

**Fig. 1:**
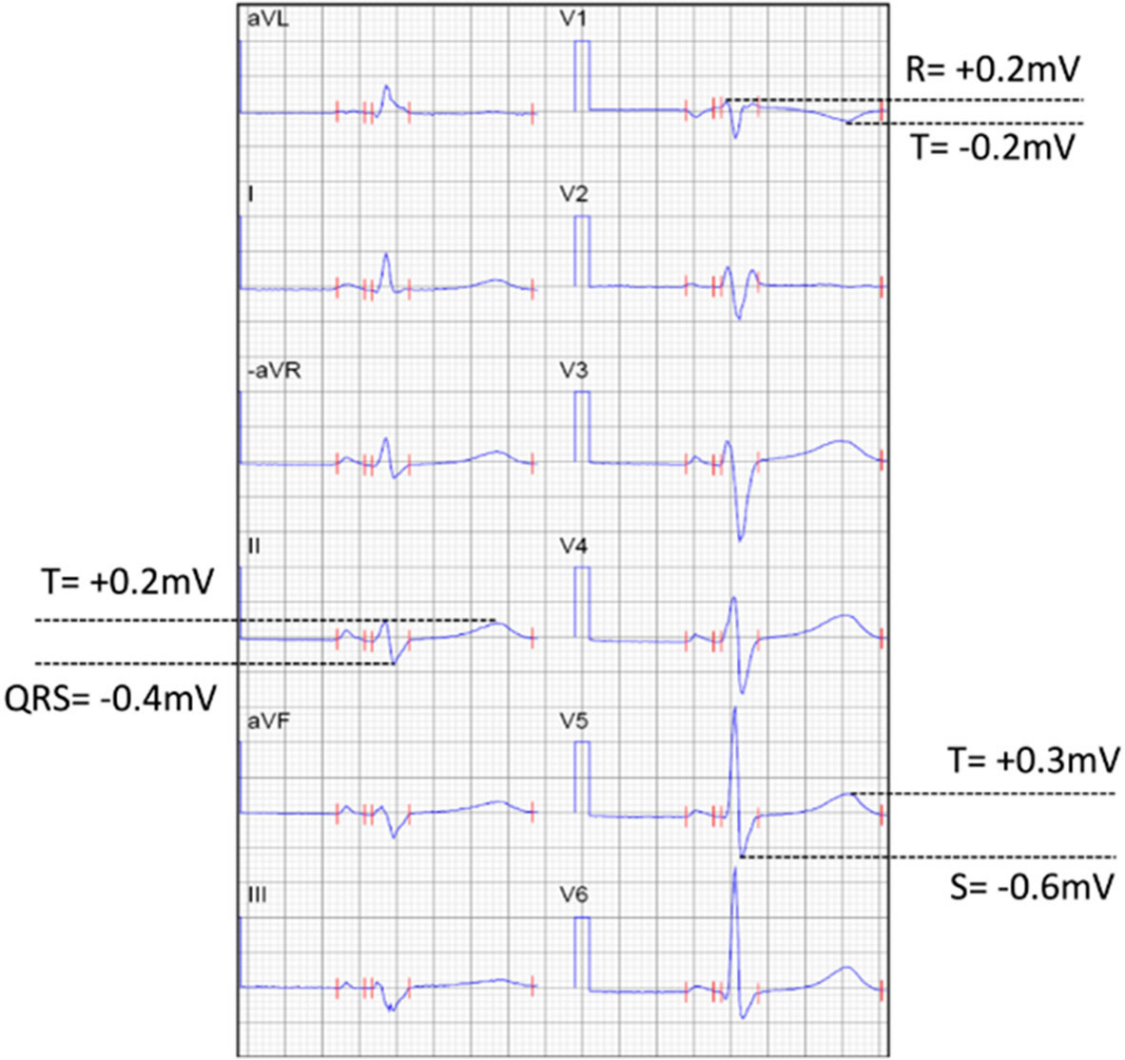
Calculation of the right spatial peaks QRS-T angle (RtSPQRS-T angle) and the right root mean square QRS (RtRMSQRS). 0.1 mV × 40 ms = 1 little box. SV5 = Swave maximum deviation from baseline (negative number). QRSmaxII: QRS maximum deviation from baseline (positive or negative number) RV1: Rwave maximum deviation from baseline (positive number). RtRMS-QRS= $\sqrt{SV{5}^2+{QRSmaxII}^2+{\left(-0.5\ast RV1\right)}^2\ }$= $\sqrt{-0.6{mV}^2+{\left(-0.4 mV\right)}^2+{\left(-0.5\ast 0.2 mV\right)}^2}$ = 0.73 mV. RtRMST=$\sqrt{TV{5}^2+{TII}^2+{\left(-0.5\ast TV1\right)}^2\ }$ = $\sqrt{0.3{mV}^2+0.2{mV}^2+{\left(-0.5\ast -0.2 mV\right)}^2}$ = 0.37 mV. RPD- angle= cos^−1^([(*SV*5∗*TV*5) + (*QRSmaxII*∗*TII*) − 0.5(*RV*1∗*TV*1)]/RtRMSQRS∗RtRMST) =cos^−1^([(−0.6*mV*∗0.3*mV*) + (−0.4*mV*∗0.2*mV*) − 0.5(0.2*mV*∗ − 0.2*mV*)]/0.73 mV∗0.37 mV) = 172.4 degrees

The spatial QRS-T angle, a vectorcardiographic parameter easily derivable from the 12-lead ECG [6], has been shown to improve detection of left sided cardiomyopathy, particularly hypertrophic cardiomyopathy [7], as well as the prediction of susceptibility to ventricular tachycardia and cardiac death both in general populations [8–10] and in patients with known cardiac pathology [11–13]. Given this mainly right-sided heart disease, we hypothesize that right-precordial-directed vectorcardiographic parameters, particularly a right precordial-directed-orthogonal QRS-T angle (RPD angle), right-sided depolarization magnitude (right root mean square of the QRS, RtRMS-QRS) (Fig. 1) from a baseline ECG would improve detection of ARVD/C patients who have no depolarization or repolarization abnormalities otherwise but who still meet criteria for definite ARVD/C by 2010 taskforce criteria (by criteria other than ECG).

## Methods

### Population

A cross-sectional study of patients with ARVC/D from an international cohort from the University of Colorado (Denver, CO, USA), Skåne University Hospital (Lund, Sweden), Linköping University Hospital (Sweden) and the University of Trieste (Italy) undergoing routine follow-up, classified as definite ARVD/C by the 2010 Task Force criteria was performed [2]. Normal variant ECGs from patients, who did not have signs of bundle branch block and not fitting 12-lead ECG major or minor depolarization or repolarization criteria by 2010 Task Force guidelines (electrocardiographically concealed ARVD/C) were compared with ECGs recorded from 1:1 age- and gender-matched control subjects who were screened in cardiology clinic at the University of Colorado (Denver, CO) or at Skåne University Hospital (Lund, Sweden) for murmurs or chest pain without family history of ARVD/C and through ultrasound and clinical observations were deemed normal. None of the control subjects had other underlying cardiac disease (no cardiomyopathy or other notable cardiac disease) nor did they have obvious obstructive or restrictive lung disease or thromboembolisms. All ECG’s were taken from the first time the patient had presented to the particular institution and no patients were on antiarrhythmic treatment at the time of their ECG. The study was approved by the Institutional Review Boards at each of the institutions noted above.

### Electrocardiogram

**Fig. 2:**
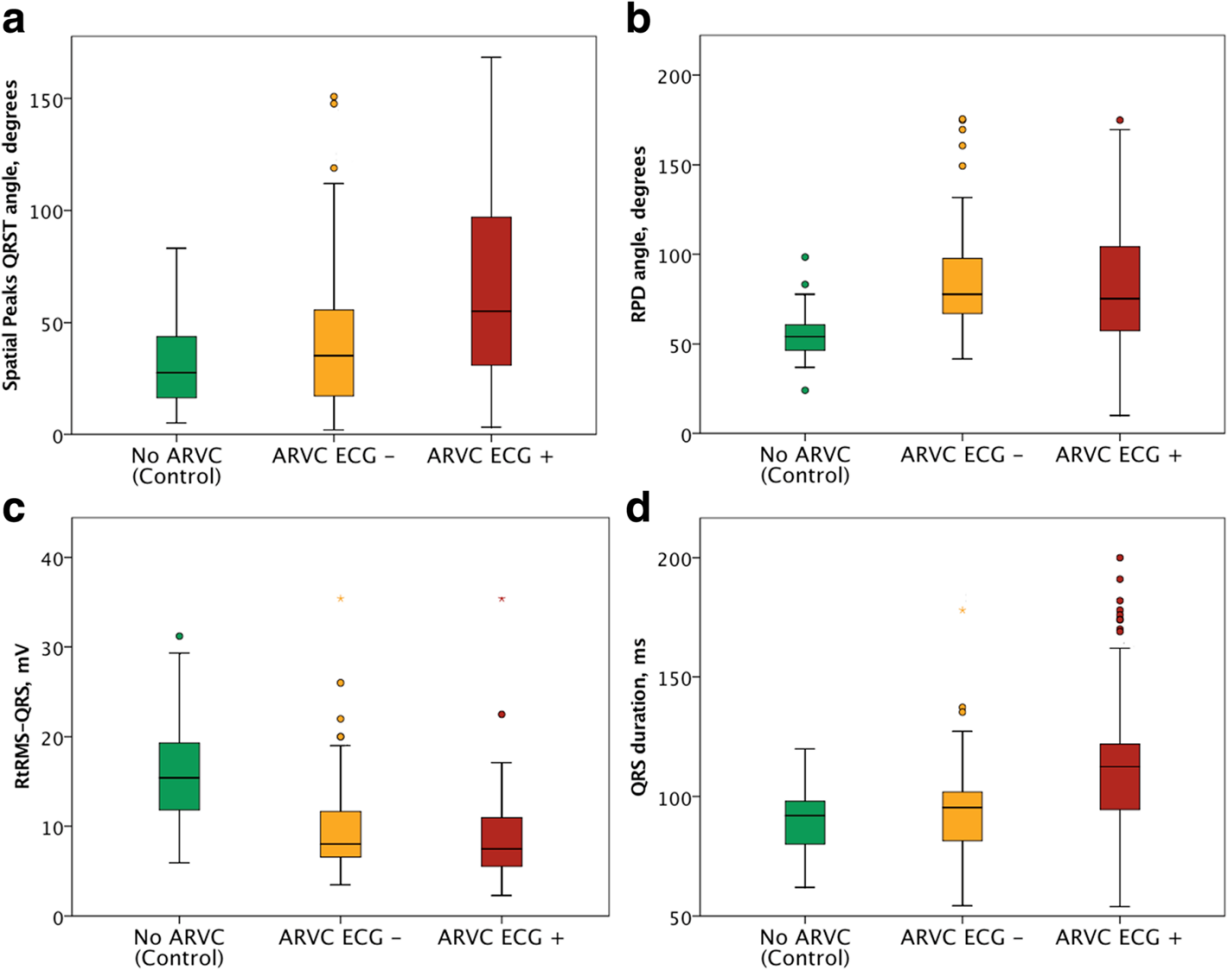
**a-d**: Box plots comparing parameter values for controls, ECG-negative and ECG-positive comparisons of the **a**: Spatial peaks QRS-T angle (median with 1st to 3rd quartiles), **b**: RPD angle (mean with 2 standard deviations), **c**: RtRMS-QRS (mean with 2 standard deviations), **d**: QRS duration (mean with 2 standard deviations)

The resting ECG closest to time of diagnostic echocardiogram or magnetic resonance imaging studies from ARVD/C patients at a speed of 25 mm/s and with voltages of 10 mm/mV were assessed (GE, WI, USA or Phillips Healthcare, MA, USA). Digital recordings were changed to PDF files and assessed at up to 150% magnification and used for vectorcardiographic derivations. Approximations of the Kors’ quasi-orthogonal spatial peaks QRS-T angle (normally based on V6 defined as the X-axis, lead II as the Y-axis and −0.5*V2 as the Z-axis) were used with direction particularly toward the R-wave in V1 (as the Z-axis QRS vector magnitude) and S-wave in V5 (as the X-axis QRS vector magnitude) while ignoring magnitudes of the S-wave in V1 and the R-wave in V5 (as an attempt to have right-precordial-directed vector magnitude and angle). Lead II measures maximum deviation from baseline (whether R or S) was used as the Y-axis QRS vector magnitude, similar to Kors’ quasi-orthogonal method [6]. Right-precordial-directed orthogonal QRS-T angles (RPD angle, degrees, Fig. 2), right-precordial-directed vector magnitudes (RtRMS-QRS, mV, Fig. 1), and spatial peaks QRS-T angles (SPQRS-T angle, degrees) were measured in ARVD/C and compared to the same parameters from control patients. The Bazett corrected QT interval (QTc) and the QRS duration (QRSd) were measured in milliseconds (ms).

The spatial QRS-T angle was calculated based on the visual transform estimation based on using selected leads and multipliers of those leads to approximate an orthogonal system. This is based on the Kors’ visual estimations regression-related method, which has been described previously [6].

The RPD angle is similar in calculation to the Kors’ quasi-orthogonal angle, but is a right-side restrictive measure meaning only the QRS maximum deviation in the orthogonal planes according to the following principles:

- X-axis: the S-wave deviation only in V5 (ignoring the R-wave in V5, even if it has a greater deviation from baseline than the S-wave);
- Y-axis: the R or S maximum deviation from lead II;
- Z-axis: the negative one half of the deviation of the R in lead V1.

These measures are then applied in the equation (Fig. 1 legend) and inverse cosine is taken between the QRS deviations and the T-wave deviations (positive or negative in leads V6 (X-axis), II (Y-axis) and negative one half of the deviation in V1 (Z-axis)). Please see Fig. 1 for further detail.

The RtRMS-QRS is the vector magnitude of the QRS complex based on right-precordial-directed measures (please see equation noted above and Fig. 1).

V5 also more consistently demonstrated an S-wave than V6, thus the S-wave in V5 was used.

All parameters were assessed by the first author if not otherwise noted above, while 10% of the sample was assessed by the 5th author to calculate inter-observer variability (per below).

### Statistics

Parametric measures are given as mean ± standard deviation, while non-parameteric measures are given as median (1st quartile to 3rd quartile) and were used to assess statistical significance between the two groups. A *p*-value of 0.05 or less was considered significant. Receiver operating characteristic curves were used to assess optimum cut-off values for sensitivity and specificity measures. Odds ratios were used. All data were de-identified. Intra-class correlation coefficients were used to determine inter−/intra-observer variability by the 1st and 5th authors’ measurements of the RPD angle, and the RtRMS-QRS.

## Results

### Population

Of a total of 155 patients with the diagnosis of definite ARVD/C by 2010 Task Force criteria, 66 patients did not have depolarization or repolarization changes consistent with either major or minor criteria (ECG-negative patients) who were compared with 1:1 age- and gender matched control patients. Tables 1 and 2 summarize patient demographic data. Apart from the ECG-related differences between ECG-positive and ECG-negative ARVD/C patients, on which group definitions were based, no other diagnostic criteria appeared to demonstrate significant difference between the groups (Table 1). Borderline significant difference was observed in regard to the greater prevalence of patients fulfilling minor arrhythmia criterion in ECG-positive ARVD/C patients.

**Table 1:** All ARVD/C, ARVD/C with (+ECG) and without (−ECG) 2010 ECG taskforce criteria

	ARVC Total (*N* = 155)	ECG-positive ARVC (*N* = 89)	ECG-negative ARVC (*N* = 66)	*p*-value ECG-positive vs ECG-negative
Age	42.1 ± 17.3	42.0 ± 17.4	41.7 ± 17.6	0.861
Sex (% male)	106 (68.4%)	63 (70.8%)	43 (65.2%)	0.488
Proband (%)	111 (71.6%)	71 (79.8%)	30 (45.5%)	0.012
I. Imaging	155 (100%)	89 (100%)	66 (100%)	1.000
major, %	111 (71.6%)	68 (76.4%)	43 (65.2%)	0.082
minor, %	89 (57.4%)	60 (67.4%)	29 (43.9%)	0.005
II. Tissue characterization of the wall, biopsies performed (% of patients)	70 (45.2%)	39 (43.8%)	31 (47.0%)	0.745
major, %	53 (75.7%)	31 (79.1%)	22 (71.0%)	0.576
minor, %	63 (90.0%)	36 (92.3%)	27 (87.1%)	0.454
III. Repolarization abnormality				
major, %	54 (34.8%)	54 (60.7%)	0 (0.0%)	<0.001
minor, %	20 (12.9%)	20 (22.5%)	0 (0.0%)	<0.001
IV. Depolarization abnormality				
major, %	13 (8.4%)	13 (14.6%)	0 (0.0%)	<0.001
minor, %	17 (11.0%)	17 (19.1%)	0 (0.0%)	<0.001
V. Arrhythmia				
major, %	44 (28.4%)	26 (29.2%)	18 (27.3%)	0.858
minor, %	64 (41.3%)	42 (47.2%)	22 (33.3%)	0.1000
VI. Family history				
major, %	84 (54.2%)	48 (53.9%)	35 (53.0%)	1.000
VII. Genotype positive	48 (31.0%)	29 (32.6%)	19 (28.8%)	0.726
PLN (% genotype positive)	1 (2.1%)	0 (0.0%)	1 (5.3%)	0.396
TTN (% genotype positive)	11 (22.9%)	8 (27.6%)	3 (15.8%)	0.488
PKP2 (% genotype positive)	23 (47.9%)	17 (58.6%)	6 (31.2%)	0.083
DSC2 (% genotype positive)	2 (4.2%)	1 (3.5%)	1 (5.3%)	1.000
DSG2 (% genotype positive)	12 (25.0%)	6 (20.7%)	6 (31.5%)	0.501
DSG3 (% genotype positive)	2 (4.2%)	1 (3.5%)	1 (5.30%)	1.000
DSP (% genotype positive)	8 (16.7%)	6 (20.7%)	2 (10.56%)	0.4510

**Table 2:** Detailed electrocardiographical and imaging characteristics of ECG-positive and ECG-negative ARVC/D patients including Epsilon waves, upslope S-wave, Signal Average ECG measurements (SAECG) including fractional QRS duration (fqrsd), low amplitude signal under 40 microV in the latter part of QRS (LAS40) and root mean square amplitude in the last 40milliseconds (RMS40), repolarization abnormalities and echocardiogram/magnetic resonance imaging (MRI) including right ventricular end-diastolic volumes (RVEDV)

	ARVD/C (*n* = 155)	ARVD/C ECG-positive (*N* = 84)	ARVD/C ECG-negative (*N* = 66)	*P*-value ECG positive/ negative
ECG: Depolarization				
- Epsilon waves	13 (8.4%)	13 (15.5%)	0 (0.0%)	<0.001
- upslope S-wave ≥55 ms V1,V2 or V3	17 (11.0%)	17 (20.2%)	0 (0.0%)	<0.001
Bundle branch blocks	22 (14.1%)	17 (20.2%)	0 (0.0%)	0.035
SAECG performed (% total)	63 (40.7%)	32 (38.1%)	31 (41.7%)	0.515
- fQRSd ≥114 ms (% of SAECG)	33 (52.4%)	17 (53.1%)	16 (51.6%)	1.000
- LAS40 ≥ 38 ms (% of SAECG)	31 (49.2%)	18 (56.3%)	13 (41.9%)	0.317
- RMS40 ≤ 20 μV (% of SAECG)	28 (44.4%)	13 (40.6%)	15 (48.4%)	0.616
ECG: Repolarization				
- T-wave inversions V1-V3 > 14 years no RBBB	54 (34.8%)	54 (64.3%)	0 (0.0%)	<0.001
- T-wave inversions V1-V4 with RBBB	12 (7.7%)	12 (14.3%)	0 (0.0%)	<0.001
- T-wave inversions V1 and V2 or in V4,V5,V6	8 (5.2%)	8 (9.5%)	0 (0.0%)	0.008
Imaging				
Echocardiograms performed (% total)	155 (100.0%)	84 (100.0%)	66 (100.0%)	1.000
- Regional akinesia/dyskinesia/aneurysm (% echo)	111 (71.6%)	65 (77.4%)	43 (65.2%)	0.108
MRI’s performed (% total)	124 (80.0%)	72 (85.7%)	52 (73.2%)	0.070
- Regional akinesia/dyskinesia/aneurysm (% MRI)	89 (71.8%)	58 (80.6%)	31 (59.6%)	0.272
- RVEDV ≥110 ml/m^2^ (M), 100 ml/m^2^ (F) (%MRI)	65 (52.4%)	49 (68.1%)	16 (30.8%)	<0.001
- RVEDV ≥100 ml/m^2^ but <110 ml/m^2^ (M), ≥90 ml/m^2^ but <100 ml/m^2^ (F) (%MRI)	24 (19.4%)	10 (11.9%)	14 (26.9%)	0.106

However, we observed significant differences in regard to the major MRI volume criteria, which was twice as common in ARVD/C patients who met ECG criteria (ECG-positive ARVD/C) than in those who did not meet 12-lead ECG criteria (ECG-negative ARVD/C). Figure 2 shows how the patients were sorted in various categories to determine the overall ability to detect ECG-negative ARVD/C patients. Figure 2a-d shows comparisons of various parameters for controls, ECG-negative and ECG-positive patients.

### QTc and QRSd

Five of our ECG-negative ARVD/C patient had right bundle branch blocks. While QRSd did not significantly differentiate ECG-negative ARVD/C patients from controls (Table 2), the QTc was significantly longer in ECG-negative ARVD/C than in control subjects (Table 3), with resultant optimum sensitivity, specificity, positive and negative predictive values shown in Table 4 with an odds ratio of 5.3 for QTc (95% confidence interval 0.6 to 46.6). The QRSd and the QTc significantly differentiated the ECG-positive versus ECG-negative ARVD/C patients (*p*-values of <0.001 and 0.002, respectively).

**Table 3:** Vector and protractor measured angles and their respective p-values for the QRS duration (QRSd, milliseconds), corrected QT interval (QTc, milliseconds), the right precordial directed angle (RPD angle), and right root mean square QRS (RtRMS-QRS)respectively for different subsets of patients including controls, arrhythmogenic right ventricular dysplasia/cardiomyopathy patients who meet 12-lead 2010 Taskforce criteria (ECG-positive), who don’t meet 12-lead 2010 Taskforce criteria (ECG-negative), who are ECG-negative without bundle branch blocks (BBB) and who are ECG-negative who have signal average ECG’s do not have any late potentials (SAECG-). *P*-values as compared to controls

Parameter	QRSd (ms),[*p*-value]	QTc (ms),[*p*-value]	SPQRS-T angle	RPD angle	RtRMS-QRS
Controls (*N* = 66)	91.5 (85.5 to 99.0)	405.0 (387.5 to 430.2)	24.1 (13.5 to 42.1)	54.4 (48.9 to 61.5)	1.54 (1.17 to 1.90)
ARVD/C ECG-positive (*N* = 89)	104.0 (94.0 to 122.0), [<0.001]*	425.0 (403.0 to 449.0), [0.022]*	43.8 (23.6 to 72.9), [0.228]	74.8 (58.4 to 94.7), [0.971]	0.81 (0.63 to 1.13), [0.371]
ARVD/C ECG- and no BBB (*N* = 66)	98.0 (86.0 to 104.0), [0.052]s	412.0 (399.0 to 430.0), [0.061]	33.6 (16.7 to 54.2), [0.004]	76.2 (62.3 to 92.9), [<0.001]	0.81 (0.64 to 1.15), [<0.001]
ARVD/C ECG-, no BBB, SAECG-negative (*N* = 20)	93.0 (85.5 to 100.0), [0.947]	420.5 (397.5 to 430.0), [0.057]	40.9 (22.3 to 55.7), [0.081]	71.2 (60.4 to 84.7) [<0.001]	0.77 (0.67 to 1.18), [<0.001]

*indicated significant *p*-value < 0.050

**Table 4:** Derived-vectorcardiographic angles and their respective sensitivities, specificities, positive and negative predictive values (PPV, NPV, respectively) and odds ratios (95% confidence intervals) for optimal cut-off values based on ROC curve analysis for the corrected QT interval (QTc), spatial peaks QRS-T angle (SPQRS-T angle), the right precordial directed angle (RPD angle angle), right root mean square QRS (RtRMS-QRS), and for both right parameters (RPD angle angle and RtRMS-QRS) at the above cut-off values) for ECG-negative ARVD/C versus controls

Parameter	Optimum cut-off	AUC	*p*-value	Sensitivity (%)	Specificity (%)	PPV (%)	NPV (%)	Odds ratio
QRSd	99.0 ms	0.64	0.026	48.5	83.3	74.4	61.8	4.7 (2.1 to 10.6)
QTc	451.0 ms	0.56	0.289	12.1	100.0	100.0	53.2	19.3 (1.1 to 342.1)
SPQRS-T angle	50.8°	0.68	<0.001	30.0	94.0	53.6	50.9	1.2 (0.5 to 2.7)
RPD angle	70.2°	0.86	<0.001	72.7	94.0	91.7	80.5	41.3 (13.1 to 130.2)
RtRMS-QRS	0.81 mV	0.85	<0.001	51.5	92.4	92.3	77.5	13.0 (4.6 to 36.4)
Both right parameters	N/A	N/A	N/A	81.8	90.9	90.0	83.3	45.0 (15.8 to 128.2)

### Spatial peaks QRS-T angles

Both the SPQRS-T angle and the RPD angle demonstrated significant differences between ECG-negative ARVD/C patients and control subjects (p-value < 0.001, Table 3). The RPD angle showed much better sensitivity and specificity than the spatial QRS-T angle (Table 4) and with an odds ratio 34 times higher than that for the SPQRS-T angle at 41.3 l95% CI 13.1 to 130.2).

The SPQRS-T angle demonstrated stepwise increase from the lowest value in the control group to ECG-negative ARVD/C and the highest value observed in the ECG-positive ARVD/C patients.

### Right root mean square QRS voltage

The right root mean square QRS (RtRMS-QRS) progressively and significantly decreased stepwise from the highest mean value observed in the control group to the lowest among the ECG-positive ARVD/C. In regard to discrimination between control subjects and ECG-negative ARVD/C patients, the ROC curve gave an optimum cut-off value of 0.81 mV giving an odds ratio of 13.0 (4.6 to 36.4). Please see Tables 3 and 4.

### Combined right-precordial directed parameters

Based on combined right-precordial-directed-sided parameters including the RPD angle and RtRMS-QRS, at the above noted cut-off values, the sensitivity, specificity and odds ratios were 90.9%, 83.3%, and 45.0 (95% CI 15.8 to 128.2), respectively. Figure 2a-d shows depolarization parameter box plots.

#### ECG-negative proband versus ECG-negative non-proband

Thirty patients without abnormalities on the 12-lead ECG were probands (45.5%). At the cut-off values above (70.2 degrees for RPD angle and 0.81 mV for the RtRMS-QRS, respectively), the sensitivity for probands was 86.7% and for non-probands 72.5% for identification of those without 12-lead ECG abnormalities otherwise, while of course maintaining specificity 92.4% and 94.0% respectively.

### ECG-based 2010 taskforce criteria and their relationship to the right-precordial ECG parameters

When the whole ARVD/C cohort was assessed (*N* = 155), the RtRMS-QRS significantly differentiated those with TAD (upslope of the S-wave ≥55 ms, minor depolarization criterion) versus those ARVD/C patients with upslope of the S-wave <55 ms (*p* = 0.006).

Patients with and without epsilon waves did not demonstrate significant difference in regard to the novel right-precordial parameters (Table 5). Patients with different extent of repolarization abnormalities, such as no T-wave inversion/T-wave inversion in V1 (repolarization criterion is not present), T-wave inversion in V1 and V2 only (minor repolarization criterion) or T-wave inversions in V1-V3 or beyond (major repolarization criterion) were not differentiated by the right-precordial parameters (Table 5). The spatial QRS-T angle was lower for those with only T-wave inversions in V1 and V2, versus those with more precordial T-wave inversions or those without T-wave inversions in the precordial leads (Table 5).

**Table 5:** Novel right-precordial and vectorcardiographic values compared to ARVD/C patients and 2010 Taskforce criteria values currently used

	SPQRS-T angle (degrees)	RPD angle (degrees)	RtRMS-QRS (millivolts)
Epsilon-wave +, *n* = 13	54.4 ± 31.3	100.5 ± 42.5	1.0 ± 0.8
Epsilon-wave -, *n* = 142	54.6 ± 42.0	81.2 ± 31.2	0.9 ± 0.4
TAD > = 55 ms, *n* = 17	67.0 ± 41.1	92.9 ± 40.7	0.7 ± 0.3^a^
TAD < 55 ms, *n* = 138	53.1 ± 41.1	81.7 ± 31.4	0.9 ± 0.5^a^
No T-wave inversion/only in V1, *n* = 26	56.7 ± 31.0	83.8 ± 33.8	1.0 ± 0.6
T-wave inversion V1-V2, *n* = 56	35.0 ± 28.0^a^	87.5 ± 31.8	0.9 ± 0.4
T-wave inversion V1-V3 or beyond, *n* = 73	67.7 ± 46.8	78.1 ± 32.4	0.8 ± 0.4

^a^indicates significantly different novel parameter values per 2010 Taskforce ECG parameter differentiation mentioned

### Left ventricular involvement and clinical parameters

Twelve total ARVD/C patients had left-sided disease (7.7%). Eleven had decreased left ventricular ejection fraction (median 52.5%, IQR 50.5 to 54.0%) and three had LVEDVi >100 ml/m^2 (median 30.8 ml/m^2, IQR 26.5 to 97.0 ml/m^2). Three patients were ECG-negative and one had no late potentials. There was only a significant difference in the QRSd with those without LV changes at median 100 ms (IQR 90-113 ms) versus those with left sided changes at a median of 97 ms (IQR 91.5 to 101 ms). The median values for ARVD/C patients with left-sided changes for the QTc, SPQRS-T angle, RPD angle and RtRMS-QRS were 417 ms (IQR 401 to 457 ms), 23.0 degrees (IQR 15.6 to 51.5 degrees), 79.4 degrees (IQR 70.0 to 99.8 degrees), and 0.91 mV (IQR 0.54 to 1.21 mV), respectively.

### Intra-observer and inter-observer variability

Intra-class correlation coefficients for the intra−/inter-observer variability for the RPD angle were 0.93 and 0.92, for the RtRMS-QRS were 0.94 and 0.92. For the SPQRS-T angle, intra−/inter-observer variability has previously been described [6, 14, 15].

Variability between automated analyses and 1st author calculations gave intra-class correlation coefficients of 0.971 and 0.917 for RtRMS-QRS and RPD angle.

### Magnetic resonance imaging correlates

MRI indexed volumes and ejection fractions were compared to the VCG/ECG parameters above. The highest R-squared value for a VCG or ECG parameter was 0.24 for the SPQRS-T angle correlating to left ventricular ejection fraction (EF). Otherwise the RPD angle and RtRMS-QRS correlated poorly to RV indexed volume (indexed RVEDV) with R-squared values at 0.15 and 0.07, respectively and with RV EF R-squared values of 0.06 and 0.19, respectively, all without significant *p*-values. QRSd also correlated poorly with indexed RVEDV and RVEF with R-squared values of 0.10 and 0.11, respectively without significant *p*-values.

## Discussion

### Main findings

We aimed to assess whether patients with definite ARVD/C diagnosed using the 2010 revised Task Force criteria exhibit subtle electrocardiographic abnormalities, which do not fit in the frame of the depolarization and repolarization criteria outlined in the Task Force 2010 document. By comparing with a cohort of healthy controls we found that ostensibly normal ECG pattern in patients with ECG-negative ARVD/C contain signs of abnormal ventricular depolarization and repolarization that can be quantified using novel right precordial-adjusted VCG markers. RPD angle, SPQRS-T angle, and the RtRMS-QRS demonstrated significant ability to differentiate patients with electrocardiographically concealed ARVD/C from healthy controls. In addition, SPQRS-T angle exhibited stepwise increase and RtRMS-QRS a decrease when control cohort was compared with ECG-negative and ECG-positive ARVD/C patients thus suggesting novel markers potential for quantification of electrocardiographic ARVD/C phenotype. These may aid in early detection in clinical cascade screening.

### QRSd and QTc

The QRSd was not a specific marker for -ECG ARVC/D, which is not surprising, given the patients don’t meet 2010 taskforce criteria including epsilon waves or delayed S-wave upstroke. It does significantly differentiate ECG-positive ARVD/C from ECG-negative ARVD/C. The QTc did significantly differentiate patients with ECG-negative ARVD/C with minimal diagnostic assistance. The QTc also prolongs significantly as the ARVD/C patients develop Taskforce 2010 ECG criteria, which may or may not assist in diagnosis.

### Spatial angles

Although conventional VCG markers have shown use in left sided heart disease [7–11], they have shown limited use in right heart disease [13]. For instance, in hypertrophic cardiomyopathy, the spatial QRS-T angle improves diagnostic ability for detection of hypertrophic cardiomyopathy over conventional 12-lead ECG parameters, however only detected part of our ECG-negative ARVD/C cohort [7]. This same angle, however showed limited prognostic ability in other right-ventricle disease patients, namely those with Tetralology of Fallot [13, 14]. The RPD angle had the highest identification ability out of all parameters tested and gave the highest odds ratio for identification of ECG-negative ARVD/C. Although the SPQRS-T angle significantly differentiates controls from ECG-negative ARVD/C, it did not prove as clinically useful with less sensitivity and less specificity than the RPD angle. Although some cases of ARVD/C include left sided disease (12 patients in our cohort), more often than not a right-sided only phenotype is present [2]. Thus, even though the SPQRS-T angle has prognostic and diagnostic use [7–11], and specifically for a generally left sided cardiomyopathy [7], it is not surprising that a right-sided specific marker is more helpful in identification of disease in those without other depolarization/repolarization abnormalities in ARVD/C as suggested by our findings. This also seemed to be particularly a good marker for those family members detected by cascade screening, who likely represent an early ARVC/D phenotype. This may be a useful marker for screening and can be programmed in most ECG software.

### Right root mean square

The RtRMS-QRS or right precordial-directed QRS vector magnitude is simply a measure of depolarization dispersion in the right ventricle which should become smaller as more fibrosis occurs. The lower the RtRMS-QRS, the more dispersion of depolarization in the right ventricle would likely occur. The RtRMS-QRS had significant identification ability in those with ECG-negative ARVD/C compared to control patients with a high specificity. This is useful as it is a simple parameter to calculate (Fig. 1). Similar to other right side-specific voltage parameters, it has low sensitivity for detection of right heart disease in this study, however as a non-invasive and cost-effective test, this simple method still detected over one half of patients who were not initially detected by ECG [15]. Given the fibro-fatty infiltration of right ventricular myocardium often observed in ARVD/C, it seems logical that dispersion of depolarization (ie. lower RtRMS-QRS) would be affected [1]. Again, this would also particularly be helpful in identification of those with early ARVC/D disease, as it was able to detect those non-proband family members who represent an early stage of ARVC/D and meet 1 of their major criteria by family association alone.

### Combined right-sided parameters

Combined, the diagnostic value of these parameters demonstrated superior identification power than each parameter alone. Combined, without compromising specificity, these parameters identified 65/71 (91.6%) of patients who would not have otherwise been identified with ECG screening. A high odds ratio was determined. These right-sided specific parameters, although not perfectly sensitive, combined have an additive identification ability without compromising specificity for patients who might otherwise fit 2010 taskforce criteria for definite ARVD/C based on genetic testing or further imaging [2].

### Novel right-precordial parameters and the degree of ARVD/C phenotype manifestation

In the case of the S-wave angle and RtRMS-QRS, there appears to be a significant step-wise progression from control patients to ECG-negative and further on to the ECG-positive ARVD/C patients, which suggests that these novel VCG/ECG markers may be considered as electrocardiographic equivalent of the disease substrate in ARVC/D. RtRMS-QRS appears to be related to the conventional electrocardiographic disease markers such as terminal activation delay in the right precordial leads, however they perform well in differentiating patients with ARVC/D from controls also in the “normal” TAD range. This demonstrates the ability of the novel VCG/ECG markers to detect ARVC/D manifesting with subtle depolarization abnormalities only and indicate their potential in identification of affected family members, which requires additional studies.

The RPD angle did not have a step-wise progression, but was similar in number between those ARVD/C patients with and those without other depolarization or repolarization abnormalities. Also, this parameter did not differentiate the degree of T-wave inversion (Table 5), thus must be more affected by depolarization versus repolarization abnormalities. Even though not a significant difference, the RPD angle (as well as the other right-precordial parameters) demonstrated trends with Epsilon wave differentiation, which seem to indicate dependence on dispersion of depolarization.

Regardless, all three parameters detect ARVD/C patients with electrographically concealed changes. Further studies are warranted to define these changes over time as well as genotype differences.

#### Limitations

The retrospective nature of this study gives inherent limitations. The study control patients were from the USA and from Sweden and did not include those from Italy, specifically, which may bias our control results to some extent. Furthermore, any type of estimation from an ECG of a parameter, if not automated carries some inherent error, although our correlation coefficients were reasonable for intra−/inter-observer variability.

## Conclusion

Patients with ECG-negative ARVD/C bear subtle ECG abnormalities that can be detected using right-sided measures including the RPD angle and the RtRMS-QRS. In combination these parameters can identify almost all patients with ECG-negative ARVD/C without compromising specificity. Future studies are warranted to identify changes in these parameters over time as well as to identify their utility in clinical cascade screening. If independently reproduced, these parameters should be considered for addition to current ARVD/C guidelines and may help to cost-effectively screen for ARVD/C in family members or those at risk.

## Abbreviations

ARVC/D: Arrhythmogenic right ventricular cardiomyopathy/dysplasia
BBB: Bundle branch blocks
DSC2: Desmocollin 2
DSG2: Desmoglein 2
DSG3: Desmoglein 3
DSP: Desmoplakin
ECG: Electrocardiogram
F: Female
fQRSd: Filtered QRS duration
ICD: internal cardiac defibrillator
LAS40: Low amplitude duration < 40 mV
M: Male
Mri: Magnetic resonance imaging
Ms.: millisecond
mV: millivolt
NPV: Negative predictive value
OR: Odds ratio
PKP2: Plakophilin 2
PLN: Phospholamban
PPV: Positive predictive value
QRSd: QRS duration
QTc: Corrected QT interval
RMS40: Root mean square voltage last 40 milliseconds of the QRS
ROC: receiver operating characteristic curve
RPD angle: Right precordial-directed angle
RtRMS-QRS: Right root mean square QRS (millivolts)
RVEDV: Right ventricular end-diastolic volume
SAECG: Signal average electrocardiogram
SPQRS-T angle: Spatial peaks QRS-T angle
TTN: Titin
